# Correction: Examining food intake and eating out of home patterns among university students

**DOI:** 10.1371/journal.pone.0207549

**Published:** 2018-11-12

**Authors:** The PLOS ONE Staff

S1 File is omitted from the list of Supporting Information. It can be viewed below. The publisher apologizes for this omission.

## Supporting information

S1 File STROBE checklist. (DOCX)
