# Supplementary material for: Examining food intake and eating out of home patterns among university students
Source: PLoS One. 2018 Oct 8;13(10):e0197874. doi: 10.1371/journal.pone.0197874 (PMC6175278; doi:10.1371/journal.pone.0197874)
Supplement: S1 Table — †Most frequently consumed foods per day (% of respondents that reported consuming an item during the recall), *Average energy contribution per day (kcal/portion size), ● = Grains and Cereals, ■ = Fruits & Vegetables, ◊ = Meat and By-Products, ♦ = Sweets and Drinks. (DOCX) [file pone.0197874.s001.docx]

**S1 Table. Frequency and mean of energy consumption of foods and drinks by source AH vs. OH by food item in Albanian students**

| **Rank** |  | **AH foods** | | | | |  | **OH foods** | | | | |
| --- | --- | --- | --- | --- | --- | --- | --- | --- | --- | --- | --- | --- |
|  |  | **%***^†^* | **Mean**  **(kcal)*** | **95%CI of mean** | | **n** |  | **%***^†^* | **Mean**  **(kcal)*** | **95%CI of mean** | | **n** |
|  |  |  |  | **Lower** | **Upper** |  |  |  |  | **Lower** | **Upper** |  |
| 1 | **●** White wheat bread | 37.1% | 383.6 | 358.8 | 256.1 | 220 | **♦** Coffee | 20.0% | 344.3 | 316.1 | 281.2 | 132 |
| 2 | **■** Apple | 4.6% | 84.4 | 79.2 | 53.9 | 124 | **●** Sandwich | 25.9% | 540.6 | 521.2 | 228.7 | 109 |
| 3 | **◊** Feta cheese | 7.0% | 134.2 | 127.6 | 72.2 | 118 | **♦** Chocolate | 8.0% | 225.8 | 208.4 | 174.7 | 81 |
| 4 | **■** Tangerine | 4.6% | 107.9 | 96.4 | 111.1 | 96 | **♦** Croissant | 5.4% | 166.9 | 148.1 | 179.8 | 74 |
| 5 | **◊** Cow’s milk | 5.6% | 162.8 | 157.5 | 64.5 | 78 | **◊** Yoghurt from shop | 11.2% | 385.1 | 369.7 | 176.3 | 66 |
| 6 | **■** Banana | 2.9% | 107.9 | 105.6 | 31.9 | 61 | **●** Bake-rolls | 4.9% | 213.2 | 201.9 | 121.4 | 52 |
| 7 | **■** Persimmons | 5.7% | 229.8 | 217.5 | 132.2 | 56 | **♦** Cola | 2.8% | 128.4 | 122.5 | 65.5 | 49 |
| 8 | **■** Tomato salad | 3.5% | 147.5 | 138.7 | 92.7 | 54 | **♦** Cookies | 5.0% | 240.3 | 222.7 | 178.0 | 47 |
| 9 | **●** Brown bread | 9.1% | 452.1 | 426.4 | 272.0 | 46 | **●** Pie with curd | 10.5% | 584.3 | 536.4 | 477.1 | 41 |
| 10 | **■** Tomato | 0.6% | 31.1 | 29.5 | 17.5 | 45 | **♦** Sugar | 0.9% | 50.1 | 46.6 | 36.1 | 39 |
| 11 | **■** Peas dish | 5.5% | 291.7 | 278.0 | 150.8 | 43 | **♦** Croissant with jam | 2.2% | 157.0 | 139.5 | 168.1 | 32 |
| 12 | **♦** Table sugar | 1.3% | 74.0 | 67.0 | 68.7 | 40 | **●** Brioche | 3.8% | 278.5 | 269.3 | 110.9 | 31 |
| 13 | **●** Spaghetti | 5.6% | 334.4 | 318.5 | 174.3 | 38 | **◊** Souvlaki | 9.8% | 797.0 | 762.7 | 385.8 | 28 |
| 14 | **●** Rice | 4.7% | 316.7 | 301.9 | 163.3 | 34 | **♦** Cake | 2.9% | 235.1 | 209.3 | 247.7 | 28 |
| 15 | **◊** Chicken roasted | 2.2% | 150.6 | 144.8 | 67.0 | 33 | **◊** Sausage | 2.0% | 175.1 | 161.7 | 134.8 | 26 |

*^†^Most frequently consumed foods per day (% of respondents that reported consuming an item during the recall)*

**Average energy contribution per day (kcal/portion size)*

● - Grains and Cereals, ■ - Fruits & Vegetables, ◊ - Meat and By-Products, ♦ - Sweets and Drinks
