# Supplementary material for: Examining food intake and eating out of home patterns among university students
Source: PLoS One. 2018 Oct 8;13(10):e0197874. doi: 10.1371/journal.pone.0197874 (PMC6175278; doi:10.1371/journal.pone.0197874)
Supplement: S2 Table — (DOCX) [file pone.0197874.s002.docx]

**S2 Table. Nutrient and energy distribution by meal**

|  | Energy (*kcal*) | Proteins (*gram*) | Fats (*gram*) | SFA (*gram*) | Cholesterol (*mg*) | Carbohydrates (*gram*) | Sugars (*gram*) | Dietary fibres (*gram*) | Sodium (*mg*) | Iron (*mg*) |
| --- | --- | --- | --- | --- | --- | --- | --- | --- | --- | --- |
| Breakfast | 477 [436.4-855.3]* | 17.1 [15.3-30.1] | 17.9 [16-31.3] | 7.4 [6.6-13] | 65.1 [55.8-109.4] | 62.4 [56.8-111.4] | 24.8 [21.8-42.7] | 4.5 [3.9-7.6] | 538.6 [468-917.2] | 3.4 [2.9-5.8] |
| Brunch | 443 [393.1-770.5] | 11.1 [9.2-17.9] | 16.5 [14.2-27.9] | 5.5 [4.6-9.1] | 30.8 [25-49] | 63.3 [56.4-110.5] | 14.2 [12-23.6] | 2.6 [2.3-4.4] | 352.1 [273.5-536.1] | 1.6 [1.3-2.5] |
| Lunch | 790.6 [741-1452.4] | 32.1 [29.5-57.8] | 35.2 [32.5-63.7] | 11.2 [10-19.7] | 74.5 [63.7-124.9] | 88.6 [81.8-160.4] | 33.5 [29.3-57.5] | 12.4 [11.1-21.7] | 779.9 [686.8-1346.2] | 6.9 [6.3-12.3] |
| Dinner | 721.4 [671.6-1316.3] | 28.3 [25.6-50.2] | 28.5 [26.2-51.3] | 10 [9-17.6] | 72.4 [61.4-120.4] | 88.9 [81.2-159.2] | 35.4 [30.6-60] | 11 [9.8-19.2] | 773 [683.4-1339.5] | 6.4 [5.8-11.3] |
| Snack | 443.1 [390.5-765.5] | 10.1 [8.3-16.2] | 12.6 [10.4-20.4] | 4.3 [3.4-6.7] | 27.1 [19.1-37.5] | 75 [66.5-130.3] | 20 [17.3-34] | 5 [4.2-8.3] | 306 [234-458.6] | 2.4 [1.9-3.7] |
| Late night meal | 324.4 [260.2-509.9] | 8.8 [5.8-11.3] | 9.4 [6.7-13] | 3.4 [2.2-4.4] | 21 [12.9-25.3] | 53.4 [44.3-86.9] | 15.1 [11.3-22.2] | 3.4 [2.6-5.1] | 248.1 [141.7-277.8] | 1.7 [1.2-2.3] |
