# Supplementary material for: Examining food intake and eating out of home patterns among university students
Source: PLoS One. 2018 Oct 8;13(10):e0197874. doi: 10.1371/journal.pone.0197874 (PMC6175278; doi:10.1371/journal.pone.0197874)
Supplement: S3 Table — %EN = Percentage of Energy. (DOCX) [file pone.0197874.s003.docx]

**S3 Table. Macronutrient content as percentage of total energy**

| **Nutrients** | **%EN** | **95% Confidence Interval of %EN** | |
| --- | --- | --- | --- |
|  |  | **Lower** | **Upper** |
| Carbohydrates AH | 51.16% | 49.37% | 52.95% |
| Carbohydrates OH | 54.76% | 52.94% | 56.58% |
| Carbohydrates Total | 51.91% | 50.89% | 52.93% |
| Proteins AH | 14.82% | 14.12% | 15.52% |
| Proteins OH | 12.39% | 11.78% | 13.00% |
| Proteins Total | 14.17% | 13.73% | 14.61% |
| Fats AH | 35.89% | 34.61% | 37.17% |
| Fats OH | 33.23% | 31.81% | 34.65% |
| Fats Total | 35.02% | 34.17% | 35.87% |
| SFA AH | 13.89% | 13.06% | 14.72% |
| SFA OH | 10.24% | 9.64% | 10.84% |
| SFA Total | 12.01% | 11.59% | 12.43% |
| Sugars AH | 12.90% | 11.62% | 14.18% |
| Sugars OH | 22.97% | 21.54% | 24.40% |
| Sugars Total | 18.22% | 17.27% | 19.17% |

*%EN: Percentage of Energy*
