# Supplementary material for: Examining food intake and eating out of home patterns among university students
Source: PLoS One. 2018 Oct 8;13(10):e0197874. doi: 10.1371/journal.pone.0197874 (PMC6175278; doi:10.1371/journal.pone.0197874)
Supplement: S1 Fig — (DOCX) [file pone.0197874.s004.docx]

**S1 Fig. Comparison of percentage of intake at home and out of home by food group**
