# Supplementary material for: Examining food intake and eating out of home patterns among university students
Source: PLoS One. 2018 Oct 8;13(10):e0197874. doi: 10.1371/journal.pone.0197874 (PMC6175278; doi:10.1371/journal.pone.0197874)
Supplement: S2 Fig — (DOCX) [file pone.0197874.s005.docx]

**S2 Fig. Nutrient composition of diet as energy percentage of total, AH and OH intake compared to WHO/FAO/UNU recommendations**

*All recommended values are based on joint WHO/FAO/UNU consultation report [1]*

1. FAO-WHO. Human Energy Requirements: Report of a Joint FAO/WHO/UNU Expert Consultation: Rome, 17-24 October 2001: Food & Agriculture Org.; 2004.
