# Supplementary material for: Examining food intake and eating out of home patterns among university students
Source: PLoS One. 2018 Oct 8;13(10):e0197874. doi: 10.1371/journal.pone.0197874 (PMC6175278; doi:10.1371/journal.pone.0197874)
Supplement: S5 Fig — (DOCX) [file pone.0197874.s008.docx]

**S5 Fig. Set 3 –Riboflavin, Thiamine, Vitamin B6, Vitamin E and Zinc**

All EU reference values are based on Regulation EU) No 1169/2011, Document No. 32011R1169 of the European Parliament ^[[1]](#footnote-1)^

1. *Can be accessed here:* [*https://eur-lex.europa.eu/legal-content/EN/TXT/?uri=CELEX:32011R1169*](https://eur-lex.europa.eu/legal-content/EN/TXT/?uri=CELEX:32011R1169) [↑](#footnote-ref-1)
