# Supplementary material for: Examining food intake and eating out of home patterns among university students
Source: PLoS One. 2018 Oct 8;13(10):e0197874. doi: 10.1371/journal.pone.0197874 (PMC6175278; doi:10.1371/journal.pone.0197874)
Supplement: S6 Fig — (DOC) [file pone.0197874.s009.doc]

**S6 Fig. Sample quantification from the booklet used in this study: (A) researcher’s copy and (B) participants’ copy**
